# Diabetes after pregnancy: a study protocol for the derivation and validation of a risk prediction model for 5-year risk of diabetes following pregnancy

**DOI:** 10.1186/s41512-021-00095-6

**Published:** 2021-03-08

**Authors:** Stephanie H. Read, Laura C. Rosella, Howard Berger, Denice S. Feig, Karen Fleming, Padma Kaul, Joel G. Ray, Baiju R. Shah, Lorraine L. Lipscombe

**Affiliations:** 1 Women’s College Research Institute, Women’s College Hospital, 76 Grenville Street, Toronto, Ontario M5S 1B2 Canada; 2 Institute for Clinical Evaluative Sciences, Toronto, Ontario Canada; 3 Evidence and Access, Certara, London, UK; 4 Dalla Lana School of Public Health, University of Toronto, Toronto, Ontario Canada; 5 Public Health Ontario, Toronto, Ontario Canada; 6 Division of Maternal-Fetal Medicine, St. Michael’s Hospital, Toronto, Ontario Canada; 7 Department of Medicine, University of Toronto, Toronto, Ontario Canada; 8 Institute of Health Policy, Management and Evaluation, University of Toronto, Toronto, Ontario Canada; 9 Sinai Health System, Toronto, Ontario Canada; 10 Department of Family and Community Medicine, Sunnybrook Health Sciences Centre, Toronto, Ontario Canada; 11 Faculty of Medicine and Dentistry, University of Alberta, Edmonton, Alberta Canada; 12 Canadian VIGOUR Centre, University of Alberta, Edmonton, Alberta Canada; 13 Department of Obstetrics and Gynaecology, St. Michael’s Hospital, Toronto, Ontario Canada; 14 Department of Medicine, Sunnybrook Health Sciences Centre, Toronto, Ontario Canada

**Keywords:** Pregnancy, Type 2 diabetes mellitus, Prognosis, Prediction model, Risk, Study protocol

## Abstract

**Background:**

Pregnancy offers a unique opportunity to identify women at higher future risk of type 2 diabetes mellitus (DM). In pregnancy, a woman has greater engagement with the healthcare system, and certain conditions are more apt to manifest, such as gestational DM (GDM) that are important markers for future DM risk. This study protocol describes the development and validation of a risk prediction model (RPM) for estimating a woman’s 5-year risk of developing type 2 DM after pregnancy.

**Methods:**

Data will be obtained from existing Ontario population-based administrative datasets. The derivation cohort will consist of all women who gave birth in Ontario, Canada between April 2006 and March 2014. Pre-specified predictors will include socio-demographic factors (age at delivery, ethnicity), maternal clinical factors (e.g., body mass index), pregnancy-related events (gestational DM, hypertensive disorders of pregnancy), and newborn factors (birthweight percentile). Incident type 2 DM will be identified by linkage to the Ontario Diabetes Database. Weibull accelerated failure time models will be developed to predict 5-year risk of type 2 DM. Measures of predictive accuracy (Nagelkerke’s *R*^2^), discrimination (C-statistics), and calibration plots will be generated. Internal validation will be conducted using a bootstrapping approach in 500 samples with replacement, and an optimism-corrected C-statistic will be calculated. External validation of the RPM will be conducted by applying the model in a large population-based pregnancy cohort in Alberta, and estimating the above measures of model performance. The model will be re-calibrated by adjusting baseline hazards and coefficients where appropriate.

**Discussion:**

The derived RPM may help identify women at high risk of developing DM in a 5-year period after pregnancy, thus facilitate lifestyle changes for women at higher risk, as well as more frequent screening for type 2 DM after pregnancy.

## Background

Type 2 diabetes mellitus (DM) is a serious metabolic condition that affects over 400 million people and accounted for 1.6 million deaths worldwide in 2016 [1]. DM and its complications are major contributors to reductions in life expectancy and quality of life [2–4]. Costs to healthcare systems attributed to DM are substantial with estimated annual global costs of US$1.3 trillion or 1.8% of global gross domestic product [5]. These costs are expected to rise further with growing type 2 DM prevalence. Some of the highest increases in DM prevalence have occurred among young women [6, 7]. Developing type 2 DM at younger ages is associated with worse morbidity and mortality compared to developing the condition at older ages [8]. Identifying opportunities for reversing the rising prevalence in young women is therefore essential to reducing the burden of DM.

Pregnancy provides a unique opportunity for estimating future type 2 DM risk, and then implementing potentially risk reducing strategies. Due to the intense physiological demands of pregnancy, some women develop temporary conditions such as GDM and hypertensive disorders of pregnancy that are important markers for future DM risk [9, 10]. GDM occurs in 5 to 20% of pregnancies and is associated with a seven-fold increased risk of type 2 DM [9–12]. Pregnancy is also a time when women are more engaged with the healthcare system and may be more motivated to implement behavioral changes to improve their health for the sake of their family.

Despite strong evidence among non-pregnant, high-risk populations that type 2 DM can be prevented with lifestyle modifications [13, 14], there remains limited post-partum follow-up relating to DM risk among women at high risk of developing DM [15]. Evidence also suggests that many women with GDM perceive their 10-year risk of developing DM to be low [16, 17]. Yet, 10–40% of women with GDM progress to DM within the first 5 years after delivery [18–20], underscoring the need for better risk communication.

A risk prediction model (RPM) for estimating future DM risk following delivery will facilitate risk communication and will support clinicians to identify women at high risk who might benefit from preventative interventions. Existing DM RPMs currently include only a small number of pregnant women and do not include important predictors of DM that develop during pregnancy [21–26]. Models developed among postpartum women are limited to women with a previous history of GDM [27–29]. These models would not be applicable to women who do not develop GDM during pregnancy but who may still be at high risk of future DM. Furthermore, each of these models were derived in small samples (*N* = ≤ 395) with limited ethnic variation.

To address the need for a population-based DM RPM that may be applied to all postpartum women, we propose the development and validation of a novel model using unique administrative datasets that capture all births within the population covered under a single-payer health system for over 200,000 deliveries.

## Methods

### Data sources

The RPM will be derived and validated using population-based administrative data collected in Ontario, Canada. These datasets are held at ICES, an independent, non-profit research institute whose legal status under Ontario’s health information privacy law allows it to collect and analyze health care and demographic data, without consent, for health system evaluation and improvement. The datasets are linked using unique encoded identifiers and analyzed at ICES.

The major source of data for this project will be Ontario’s perinatal registry, the Better Outcomes Registry and Network (BORN). This dataset was established in 2009 and captures information from hospitals, midwifery practice groups, specialized antenatal clinics, prenatal screening laboratories, and fertility clinics for all deliveries occurring within hospitals in Ontario. Data quality of BORN data elements was recently assessed and was found to have good agreement with data from patient charts [30]. Of the 29 data elements assessed, 23 elements had greater than 90% agreement, including maternal weight and height. Prior to BORN, pre- and peri-natal data were collated in the Niday database, now known as the BORN legacy data [31]. This earlier database was first created in 1997 and by 2008 captured data for 96% of deliveries in Ontario. While data quality for Niday data is lower, the percentage agreement between Niday and patient chart for important predictors including infant birthweight and gestational age at delivery exceed 90% [31]. Data collated in both databases include maternal demographic, behavioral, and health status characteristics. Obstetric complications and delivery outcomes are also captured. BORN is funded by the Ontario Ministry of Health and Long Term Care and administered by The Children’s Hospital of Eastern Ontario. Since the administrative data do not capture information on ethnicity, maternal ethnicity will be ascertained using a validated algorithm which uses surnames to assign an ethnicity (South Asians, Chinese, Other) to all residents of Ontario [32]. Linkage to the Ontario Laboratories Information System (OLIS) will be used to capture 50 g oral glucose challenge test (OGCT) values. OLIS was established in 2006 and holds data relating to medical laboratory test orders and results from community, hospital and public laboratories across Ontario. The OGCT is used to screen for GDM, is currently offered to all pregnant women and is typically administered between 24 and 28 weeks’ gestation [33]. Data relating to comorbidities such as cardiovascular disease and prior pregnancy history will be obtained from the Discharge Abstract Database (DAD). This database captures detailed clinical and administrative data for hospital admissions and day surgeries in Ontario.

To obtain outcome data, we will use the Ontario Diabetes Database (ODD). This database was established in 1991 and contains all individuals with DM in Ontario. This database captures data from hospital discharge abstracts, Ontario drug benefit claims and physician service claims and data from this database are currently available until 31st March 2019. The use of data in this project was authorized under section 45 of Ontario’s Personal Health Information Protection Act, which does not require review by a Research Ethics Board.

### Participants

The derivation cohort will consist of all women aged between 16 and 50 years (at index pregnancy) whose pregnancy resulted in a live birth or still birth in Ontario between 1st April 2006 and 31st March 2014. No minimum duration of pregnancy was required for inclusion into the derivation cohort. For women who had multiple pregnancies during this period, the first pregnancy occurring during the accrual window will be selected. The influence of this choice of pregnancy will be examined in sensitivity analyses involving the development and validation of models in separate cohorts containing a randomized choice of pregnancy. Women with pre-pregnancy DM as ascertained using the ODD and available variables in BORN indicating pre-existing DM will be excluded from the derivation cohort. Women who were ineligible for the Ontario Health Insurance Plan or were a non-Ontario resident in the 2 years prior to index date will be excluded since predictor and outcome data for these women will be incomplete. Index date is defined as 6 months post-partum. Women who had a second pregnancy prior to the index date and women who died prior to index will also be excluded.

### Main predictors

Table 1 lists the predictors and the pre-specified functional forms for each.

**Table 1:** Pre-specification of predictors for risk prediction model

Variable type	Variable	Specification	df
Demographics	Age at index	Continuous variable	1
	Income quintile	Five categories	4
	Rurality	Binary: yes/no	1
	Immigrant status	Three categories (non-immigrant, previous (over 10 years ago), recent (10 years or less)	2
	Ethnicity	Three categories (South Asian, Chinese, Other)	2
Lifestyle	Pre-pregnancy BMI, kg/m^2^ (self-reported)	Continuous	1-3
Lifetime Health history	Previous mental health condition	Binary: yes/no	1
	Previous cardiovascular disease	Binary: yes/no	
Prior pregnancy history	Parity	Five categories (0, 1, 2, 3, > 3)	4
	Gravidity	Four categories (1, 2, 3, > 3)	3
	Previous GDM	Three categories: yes/no/NA	2
	Previous gestational hypertension or preeclampsia	Three categories: yes/no/NA	2
Index pregnancy	50 g oral glucose challenge test, mmol/L	Continuous	1-3
	GDM	Binary: yes/no	1
	Preeclampsia	Binary: yes/no	1
	Gestational hypertension	Binary: yes/no	1
	Caesarean-section	Three categories: planned/emergency/no	1
	Premature delivery	Binary: yes/no	1
Newborn	Birthweight	Continuous (for women with a multi-fetal birth, the child with the higher birthweight will be used)	1
	Large for gestational age (LGA) birthweight > 90th	Binary: yes/no (for women with a multi-fetal birth, any child with a record for LGA will be used)	1

Choice of the candidate predictors was informed by a systematic literature review, consultation with the project advisory board, clinicians, and the availability of the predictors within the study’s data sources. To enhance usability, predictors of the model were also chosen based on their likely availability to intended users of the model. Continuous variables with limited variation and binary variables with small counts will be excluded. While categorical variables have been pre-specified, frequency distributions will be examined and categories may be combined where there are small numbers. Interactions between all predictors and each of age, GDM and ethnicity will be considered for inclusion into the model due to expected differences in the effect of these predictors by the selected variables. Interactions which improve model fit will be included in the final model.

### Outcome

Women will be followed-up for the incidence of type 2 diabetes. The incident onset of type 2 DM will be ascertained using a validated definition [34]. That algorithm requires one hospital record including a diabetes-specific International Classification of Disease code (ICD) OR two physician claim records relating to diabetes treatment within 1 year of each other, OR a dispensing record for an anti-diabetic drug from the Ontario Drug Benefit. According to this definition, diabetes records occurring between 120 days before or 180 after a hospital or primary care record of pregnancy care are considered to relate to gestational diabetes and are excluded.

Where physician claim records are used to ascertain DM status, DM diagnosis date is set to the date of the first visit. The validated definition has a sensitivity of 90.0%, a specificity of 97.7%, and a positive predictive value of 82.6%. Women will be followed up from index date until the earliest of incident diabetes, death, new pregnancy conception date, or study end-date (31st March 2019).

### Sample size

For the period between 2014 and 2016, there were 231,618 women who delivered a baby and 756,956 person-years of follow-up. Overall, 2294 women developed incident type 2 DM during follow-up (unpublished data). Using the criteria specified by Riley et al. and the *pmsampsize* R package, we calculated the minimum sample size required to be 6193 women to minimize overfitting and ensure the estimation of precise model coefficients [35, 36].

### Analysis plan

All data manipulations will be carried out using Wickham’s *tidyverse* package [37] and modelling will be conducted using Harrell’s *rms* package in R [38]. The TRIPOD statement was used to devise the analysis plan and will be used to guide the reporting of the development and validation of the proposed model [39].

### Data cleaning and coding of predictors

Continuous predictors will be assessed using descriptive summaries and histograms. Implausible values will be set to missing, and highly skewed predictors will be truncated at the 99.5 percentile. For example, if a woman has a pre-pregnancy BMI value of 51 kg/m^2^, which surpasses the calculated 99.5^th^ percentile BMI of 50 kg/m^2^, then her BMI will be assigned as 50 kg/m^2^. Continuous predictors will be centered on their mean values. Continuous variables will be included in the model as linear or non-linear terms using restricted cubic splines, depending on model fit. Knot placement in restricted cubic splines will be based on the percentile distribution of the continuous variable. The definitions of all variables have been pre-specified to minimize risk of over-fitting.

### Missing data

Predictors with missingness exceeding 40% will be excluded from the RPM [40]. Missing data among the remaining predictors will be assumed missing at random, conditional on the available variables, and will be addressed using multiple imputation [41, 42]. Logistic regression models will be used to identify predictors of missingness that should be included in the imputation model. To identify the likely ability of the imputation model to accurately impute missing data, we will conduct exploratory analyses to examine associations between variables with missing data and available predictors.

The imputation model will contain all time to event, censoring, predictor, and auxiliary variables [43]. Predictor variables will be included in the imputation model in the same functional form as they will appear in the pre-specified prediction model (i.e., continuous, categorical). Auxiliary variables which can provide information on the missing values will be included to improve the accuracy of the imputations. For example, to facilitate the imputation of BMI, pre-pregnancy weight will be included in the imputation model as an auxiliary variable. The *mice* package in R will be used to generate 10 imputed datasets. The model will be generated in each imputed dataset and combined according to Rubin’s rules [41].

At model deployment when the outcome is unknown and when predictors may be unavailable, it will be necessary for the tool to impute missing predictor data in real-time using single or multiple imputation approaches, where feasible. To emulate multiple imputations in this setting, the model’s performance will be assessed in two sets of imputed datasets. The first set of imputed datasets will be derived using imputation models that include the outcome variables while the second set will be derived in imputation models that exclude the outcome variables. The assessment of model performance in the second dataset will more accurately describe likely performance at model deployment [44]. Should multiple imputations at deployment not be feasible, single imputation methods will be explored. The chosen single imputation approach for handling missing data at model deployment will be replicated during external validation to obtain an accurate assessment of likely model performance at deployment.

### Model estimation

Model coefficients will be estimated using Weibull accelerated failure time models to calculate 5-year risk of type 2 DM. This parametric model was chosen since it allows for the calculation of more clinically meaningful parameters, including predicted survival time for different follow-up periods.

A full model, including all predictors and important interactions, will be developed in the first instance. Since the practical application of the full model may be time-consuming for intended users, we will derive a less complicated model by applying Ambler’s step-down approach [45]. This approach involves the regression of the derived prognostic index from the full model in the predictors. Predictors are subsequently dropped that produce the smallest reduction in *R*^2^. This procedure will be repeated until the exclusion of any further predictors would lead to a *R*^2^ value below 0.95. This approach will ensure that variables that contribute very limited information to the model are removed. We will verify our model building approach in exploratory analyses by applying least absolute shrinkage and selection operator (LASSO) to conduct variable selection and regularization.

### Assessment of RPM discrimination

Discrimination, which describes the models ability to distinguish between women who did and did not develop type 2 DM, will be assessed using C-statistics. C-statistics will be calculated at various time points (e.g., 1 year, 2 years, 5 years). The clinical usefulness of the RPM will be quantified using the net benefit approach [46].

### Assessment of RPM calibration

Overall model calibration describes the agreement between observed and predicted risks. Calibration will be assessed using calibration plots and estimation of calibration slopes and calibration-in-the-large values in each imputed dataset. Calibration curves will be generated in each imputed dataset and combined into a single plot. Calibration slopes will be estimated at fixed time-points by regressing the observed risk of type 2 DM on the predicted prognostic index. Calibration-in-the-large will be estimated by comparing the mean observed risk estimated using the Kaplan-Meier method with the mean predicted risk. Calibration will be assessed within predefined groups, including by GDM status, ethnicity and age. Formal statistical testing of calibration using Hosmer-Lemeshow goodness of fit tests will not be performed, due to the large sample size.

### RPM predictive accuracy

Overall predictive accuracy will be assessed with the Brier score and by estimating explained variation using Nagelkerke’s *R*.

### RPM internal validation

To assess the degree of optimism in the estimated performance, internal validation using a bootstrapping approach, with 500 resamples will be performed. Using this approach, the model will be derived in each of the 500 bootstrap samples. Each of the bootstrap sample models will then be applied within the original dataset and measures of performance will be calculated for each bootstrap sample model. The difference in model performance between bootstrap sample models and the original model will be used to estimate the optimism corrected measures of performance, including Nagelkerke’s *R*^2^ and C-statistics. Over-fitting will be quantified by the calculation of a uniform shrinkage factor. Where necessary, the uniform shrinkage factor will be used to adjust the mode coefficients.

### Model presentation

The final regression model and validation results will be published in full in a peer-reviewed journal and according to the TRIPOD guidelines. The regression formula will be subsequently incorporated into a web-based calculator and will be integrated into electronic healthcare records. By integrating a calculator into electronic healthcare records, general practitioners will be able to readily access the necessary input data to estimate diabetes risk in postpartum women.

### External validation

We will externally validate the algorithm using data from a population-based pregnancy cohort from Alberta, the Alberta Vital Statistics–Birth database [47]. This database is populated by maternal, pregnancy, and neonatal data and is linked to hospital admissions records (Discharge Abstract Database), emergency room/outpatient clinic visits records (the Ambulatory Care Classification System (ACCS)), and physician office visit records (Fee-for-Service Claims (CLAIM)). Linkage to the Alberta Health Care Insurance Population registry provides demographic data including ethnicity and rurality. Diabetes will be ascertained using ICD-9 and ICD-10 codes in hospitalization, ACCS, or CLAIM records. The RPM will be applied in this cohort to estimate 5-year risk of RPM in this external population. The predictive performance of the RPM will be assessed by estimating the previously described measures of calibration and discrimination. Where appropriate, the RPM model will be re-calibrated by adjusting the baseline hazard and the mean values of the predictors to that of the external validation cohort [48].

## Discussion

We have described a protocol for the development and validation of a novel RPM using a large population-based cohort derived from validated databases. This will be the first RPM derived for use among all women following pregnancy and not just for those that developed GDM. The RPM may be embedded in electronic health records to help to guide clinical decision-making in family practices and to communicate risk. A web-based calculator will also be generated to enable women to calculate their own risk of developing diabetes and potentially motivate them to make lifestyle changes. The development of the web-based calculator will be informed by data collected during a qualitative study to identify optimal approaches to communicating type 2 diabetes risk among post-partum women.

### Limitations

A key limitation of this study will be our inability to be certain about a woman having type 2 DM vs. type 1 DM; therefore, the minority of diagnosed with type 1 and type 2 DM following pregnancy will be included as an outcome event, however unlikely. These women are therefore unlikely to benefit from postpartum lifestyle preventative interventions. However, since approximately 90 to 95% of diagnosed type 2 DM cases are type 2 DM and this number increases with increasing age at diagnosis, only a very small number of subsequent type 2 DM cases are likely to be type 1 DM. A second limitation is the presence of missing data in the derivation cohort. We will address this problem by only including predictors that are at least 60% complete and by using multiple imputations, a widely accepted approach to handling missing data. Some predictors, such as OGCT values are unlikely to be available for every woman since OLIS does not capture results from all laboratories across the province. Since the OGCT is offered to all pregnant women and screening rates are high in Canada [49], missing data in this variable should predominantly relate to whether the attending site was contributing submissions to OLIS. Given that evidence suggests no differences in characteristics of people attending OLIS contributing and non-contributing sites [50], it may be reasonable to assume that these data are missing completely at random, in which case multiple imputation is an appropriate missing data handling approach [41]. However, exploratory analyses will be undertaken to determine whether OGCT values can be reasonably imputed using available data.

Thirdly, while we intend to validate our RPM in a temporal validation cohort consisting of women with GDM, it will be necessary to further assess the generalizability of the model to other non-overlapping populations of postpartum women. Finally, we do not have data relating to family history of diabetes, an established predictor of type 2 diabetes risk. However, it is unknown to what extent family history of diabetes predicts risk of diabetes over and above the available predictors, specifically oral glucose tolerance test values.

## Conclusion

The global burden of type 2 DM is increasing and identifying opportunities to reduce risk of type 2 DM is a key priority. Pregnancy offers one such opportunity due to increased healthcare engagement and the development of conditions associated with type 2 DM risk. An accurate type 2 DM RPM may be used by clinicians to identify women at greatest risk and who would benefit from lifestyle interventions. Our proposed model will be the first type 2 DM RPM for use among all pregnant women. It will be derived in a diverse population using large administrative data sources and will be used to enhance post-partum care of women at high risk of developing type 2 DM.

## Data Availability

The data that support the findings of this study are available from ICES and the Manitoba Centre for Health Policy, but restrictions apply to the availability of these data, which were used under license for the current study, and so are not publicly available. Data are however available from the authors upon reasonable request and with permission of the Institute for Clinical Evaluative Sciences or the Manitoba Centre for Health Policy. Where possible, the analytic code will be uploaded to a free, open platform.

## Abbreviations

ACCS: Ambulatory Care Classification System
BMI: Body mass index
BORN: Better Outcomes Registry and Network
CI: Confidence intervals
DM: Diabetes mellitus
GDM: Gestational diabetes mellitus
ICD: International Classification of Disease
ODD: Ontario Diabetes Database
OHIP: Ontario Health Insurance Plan
OLIS: Ontario Laboratories Information System
RPM: Risk prediction model
TRIPOD: Transparent reporting of a multivariable prediction model for individual prognosis or diagnosis
